# A new species of *Hyalella* from the High Andes of Ecuador (Crustacea, Amphipoda, Hyalellidae)

**DOI:** 10.3897/zookeys.686.12223

**Published:** 2017-07-24

**Authors:** Miguel Alonso, Damià Jaume

**Affiliations:** 1 Departamento de Recursos Hídricos y Ciencias Ambientales, Facultad de Ciencias Químicas, Universidad de Cuenca, Cuenca, Ecuador; 2 Ecology Section, Department of Evolutionary Biology, Ecology and Environmental Sciences, Faculty of Biology, University of Barcelona, Avda. Diagonal 643, 08028 Barcelona, Spain; 3 IMEDEA (CSIC-UIB), Mediterranean Institute for Advanced Studies, C/ Miquel Marquès 21, 07190 Esporles, Illes Balears, Spain

**Keywords:** Crustacea, descriptive taxonomy, high altitude lakes, *Hyalella*, new species, South America

## Abstract

*Hyalella cajasi* **sp. n.** is described from high altitude shallow water lakes in southern Ecuador. This is the second representative of the genus recorded in the country after *H. meinerti*. The new species shares with nine South American species of the genus the display of a smooth, non-processiferous body, a male first uropod with a modified curved robust seta on the endopod, and six pairs of sternal gills. The new taxon can be distinguished from these species based on the presence/absence of eyes; relative length of antenna 1 with respect to antenna 2; presence/absence of short pointed robust seta distally on palp of maxilla 1; number of pappose setae proximally on medial margin of inner lobe of maxilla 2; elongation and curvature of the modified robust seta of endopod of male uropod 1; relative length of ramus of uropod 3 with respect to protopod; and armature and outline of telson, among other features. It seems to be a high-altitude endemic to the Cajas Massif in Azuay Province, being replaced in the same area at lower altitudes by *H. meinerti*.

## Introduction

*Hyalella* Smith, 1874 is the only genus of epigean freshwater amphipod known to occur in South America. The genus is the single member of the family Hyalellidae Bulyčeva, 1957, endemic to the Nearctic and Neotropical regions, where it is broadly distributed and diversified, to a current total of about 70 species (Horton and Lowry 2013). The highest diversity is attained in Brazil, which harbours 22 species, although Lake Titicaca with 17 species –15 of which are endemic– concentrates the highest number relative to a restricted area. Here we describe *Hyalella cajasi* sp. n., a high altitude species found at Cajas National Park, near the city of Cuenca (southern Ecuador). It represents the second taxonomically substantiated record for the country after *H. meinerti* Stebbing, 1899, recorded by González and Watling (2003) at 1,500 m above sea level (a.s.l.) on the road between Guayaquil and Cuenca, thus close to the location of the new species. Other species records from Ecuador are not adequately substantiated and should not be considered in faunistic or biogeographic analyses. These include those of *H. inermis* S. I. Smith, 1875, a species known only from Colorado, U.S.A., reported by Whymper (1892), and H. cf. dentata reported by Gunkel and Beulker (2009), a taxon currently considered to be a junior synonym of the North American *H. azteca* (Saussure, 1858) (see Horton and Lowry 2013).

## Materials and methods

Sampling was carried out during limnological surveys of 202 water bodies (lakes, lagoons and ponds) located between 3,150 and 4,460 m a.s.l. at Cajas National Park (Southern Ecuadorian Andes), in the framework of the project “Limnological Characterization of the lakes and lagoons of Cajas National Park”, funded by the University of Cuenca (Ecuador) and by Empresa Pública Municipal de Telecomunicaciones, Agua Potable, Alcantarillado y Saneamiento (ETAPA), of the municipality of Cuenca.

Samples were collected in 2015 in the littoral zone directly with a hand-held plankton net and fixed, *in situ*, in 95% ethanol. Once in the laboratory, specimens were dissected in lactic acid under the stereomicroscope, and appendages illustrated using a Leica DM2500 microscope equipped with Nomarski differential interference contrast and a drawing tube. Body measurements were derived from the sum of the maximum dorsal dimensions of body somites and exclude telson length. Type material is deposited in the Museo Ecuatoriano de Ciencias Naturales del Instituto Nacional de Biodiversidad, Quito, Ecuador [MECN].

The new species is known only from high-altitude (3,859 to 4,103 m a.s.l.) lacustrine water bodies at Cajas National Park, in the southern Ecuadorian Andes, where it is apparently endemic. It occurs both in the littoral zone of lakes and in shallow lagoons and temporary ponds. These water bodies are oligotrophic and low mineralized (Conductivity: 9.84-91.50 µS/cm), although they carry a significant amount of dissolved organic carbon (DOC) derived from adjacent terrestrial ecosystems, mainly Páramo grassland (“Pajonal”).

## Taxonomy

### Order Amphipoda Latreille, 1816

#### Family Hyalellidae Bulyčeva, 1957

##### Genus *Hyalella* S. I. Smith, 1874

###### Hyalella cajasi sp. n.

Taxon classificationAnimaliaORDOFAMILIA

http://zoobank.org/7C4E336D-2489-45DF-8056-407428866AEE

**Figure 1.**
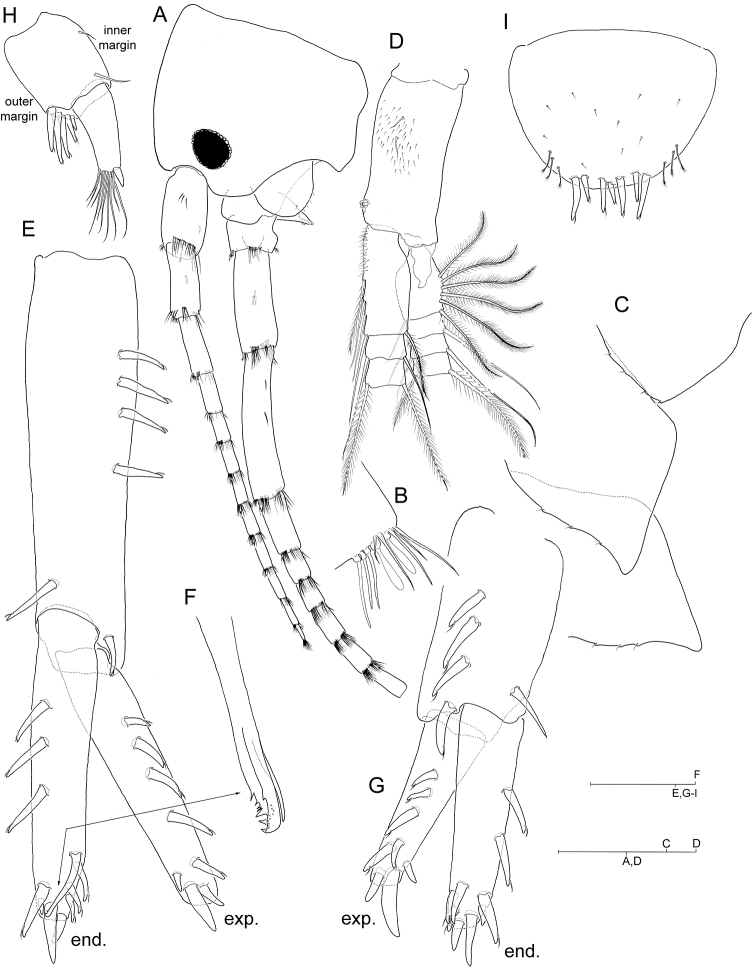
*Hyalella cajasi* sp. n., male. **A** head with left antenna 1 and antenna 2 attached, lateral **B** inset of distomedial armature on posterior margin of articles 3-6 of antenna 1 **C** right epimeral plates **D** right pleopod 3, posterior. Scale bars: 0.2 mm (**A, D**); 0.1 mm (**B**); 0.5 mm (**C, E, G–I**); 0.05 mm (**F**).

**Figure 2.**
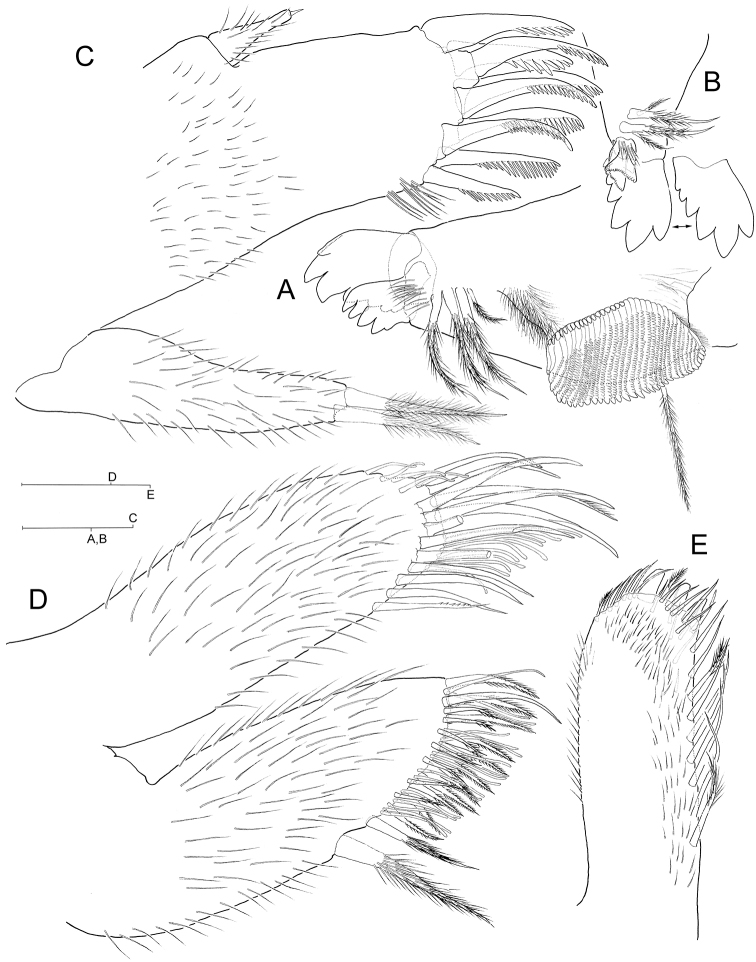
*Hyalella cajasi* sp. n., male. **A** left mandible **B** distal portion of right mandible **C** maxilla 1 **D** maxilla 2 **E** inset of basal endite (= inner plate) of left maxilliped, anterior view. Scale bars: 0.1 mm.

**Figure 3.**
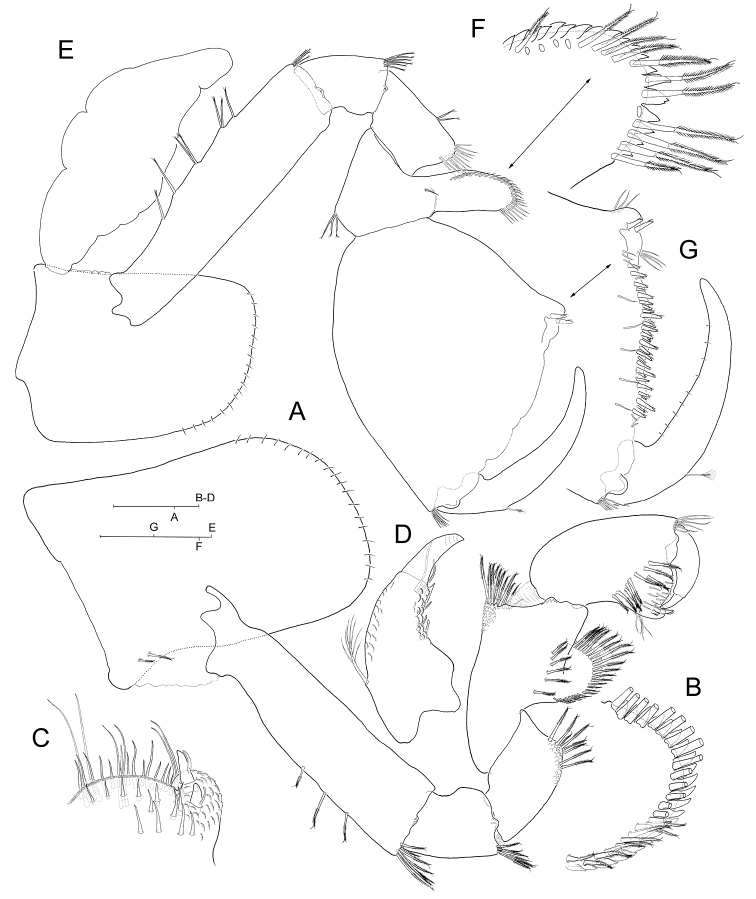
*Hyalella cajasi* sp. n., male. **A** left gnathopod 1, medial (armature on palm margin of propodus and on dactylus omitted) **B** inset of carpal lobe, medial **C** inset of palm margin and palm angle of propodus, medial **D** inset of dactylus, medial **E** right gnathopod 2, medial (armature on palm margin of propodus and on dactlylus omitted) **F** inset of carpal lobe, medial **G** detail of palm margin and palm angle of propodus, and of dactylus, medial (submarginal armature along lateral side of palm margin omitted). Scale bars: 0.2 mm (**A, G**); 0.1 mm (**B–D, F**); 0.5 mm (**E**).

**Figure 4.**
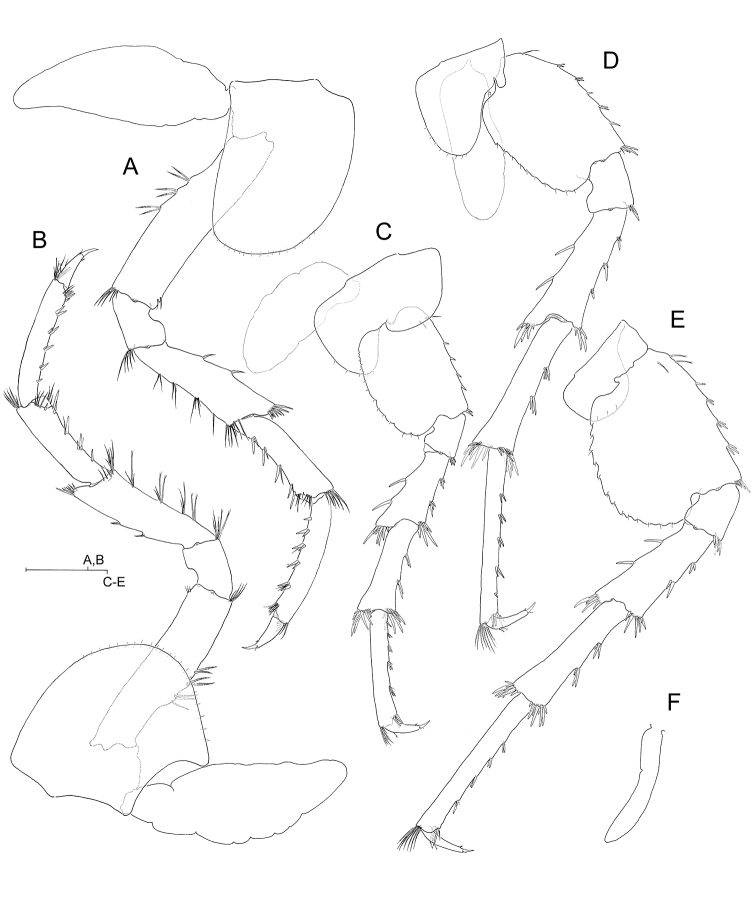
*Hyalella cajasi* sp. n., male. **A** right pereiopod 3, lateral **B** right pereiopod 4, lateral **C** right pereiopod 5, lateral, coxal gill omitted **D** right pereiopod 6, lateral, coxal gill omitted **E** left pereiopod 7, medial **F** left sternal gill on sternite of pereionite VII. Scale bars: 0.5 mm.

**Figure 5.**
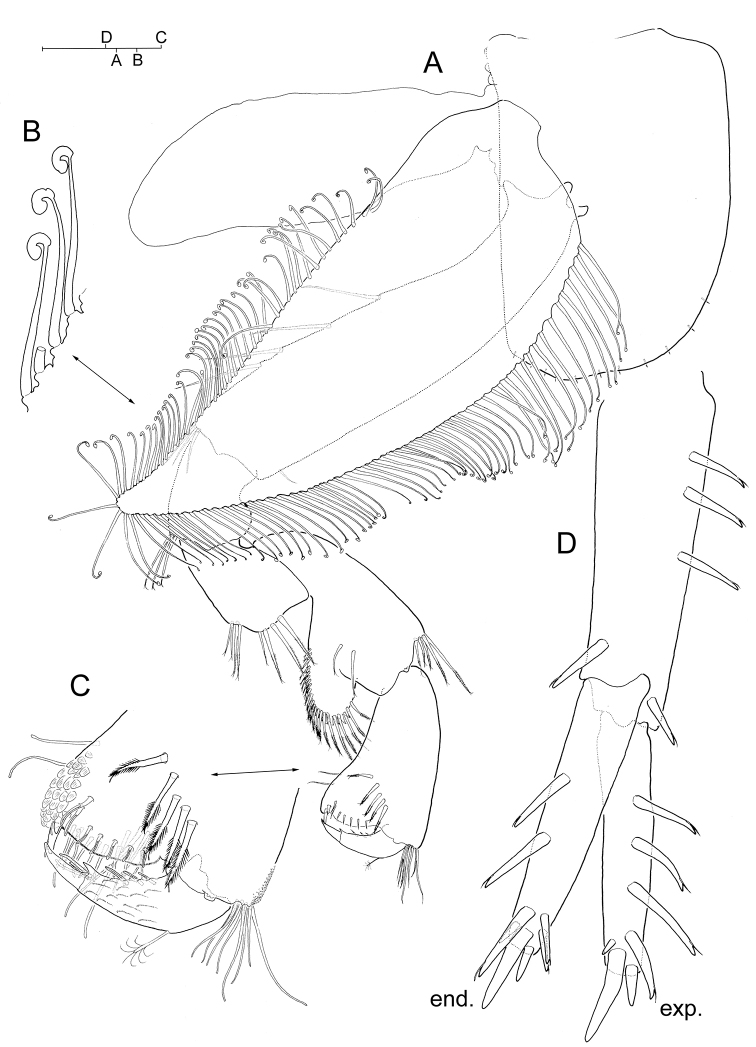
*Hyalella cajasi* sp. n., female. **A** left gnathopod 2, medial **B** inset of marginal setae on oöstegite **C** inset of distal margin of propodus plus dactylus, medial **D** right uropod 1, posterior. Scale bars: 0.2 mm (**A**); 0.05 mm (**B**); 0.1 mm (**C, D**).

Figs 1 , 2 , 3 , 4 , 5

####### Material examined.

All collected by Henrietta Hampel at Cajas National Park (Azuay Province; southern Ecuador), 5^th^ May 2015. Laguna Togliacocha (S2°47'55.90"; W79°15'02.13"); 3,859 m a.s.l. HOLOTYPE: male 9.0 mm, preserved in formaldehyde vial. PARATYPES: Five males and 5 females in formaldehyde vial. Holotype and paratypes registered under same registration number [MECN-SI-Cal-0003]. Laguna Cardenillo (S2°46'54.32"; W79°14'50.48"); 4,103 m a.s.l. Four males and 6 females. Small laguna between Laguna Cardenillo and La Negra (S2°47'04.21"; W79°14'50.90"); 4,076 m a.s.l. Nine males and 8 females. Laguna Azul (S2°47'17.81"; W79°14'46.19"); 4,043 m a.s.l. Five males and 5 females. Small laguna close to La Larga (S2°47'35.70"; W79°14'44.08"); 3,954 m a.s.l. Ten males and 9 females. Pool close to Laguna Luspa (S2°47'54.04"; W79°15'46.94"); 3,868 m a.s.l. Ten males and 10 females. Laguna Illincocha (S2°46'46.59"; W79°13'51.33"); 3,986 m a.s.l. One male and 4 females.

####### Diagnosis.

Body smooth, non-processiferous. Eyes normal. Antenna 1 much shorter than antenna 2. Incisor of mandibles multi-denticulate. Palp of maxilla 1 short, reaching to less than half distance between its insertion and distal margin of outer lobe, crowned with short robust seta. Inner lobe of maxilla 2 with two pappose setae proximally on medial margin. Coxal plate IV deeply excavated posteriorly. Six pairs of sternal gills, on pereionites II to VII. Uropod 1 sexually dimorphic, with male exhibiting a modified robust seta (“copulatory spine” *sensu* Bousfield 1996) on endopod; modified robust seta hardly curved and only just surpassing tip of endopod. Ramus of uropod 3 shorter than protopod. Telson broader than long, with distal margin evenly rounded; distal armature arranged as a single row of robust setae.

####### Etymology.

Species name refers to Cajas National Park (Azuay Province; southern Ecuador), the type locality.

####### Description of male.

***Body*** (not figured) up to 9.0 mm long, strongly pigmented, virtually black, smooth. ***Head*** (Fig. 1A) shorter than pereionites I and II combined; rostrum wanting; lateral lobes truncate; postantennal sinus shallow and broad. Eyes small, round, located behind insertion of antenna 1. ***Epimeral plates*** (Fig. 1C) each with unarmed distal margin; plates II–III posterodistally acuminate with posterodistal angle strongly produced, subacute; posterior margin of plates with several tiny tooth-like projections each provided with short setule.

***Antenna 1*** (Fig. 1A) much shorter than antenna 2 although longer than peduncle of latter; peduncle longer than head, segments 1–3 relative length as: 1: 1: 0.8; main flagellum longer than peduncle; accessory flagellum absent. Pair of aesthetascs present on distomedial angle of posterior margin of middle articles of flagellum (Fig. 1B).

***Antenna 2*** (Fig. 1A) peduncle segments 4–5 relative length as: 0.7: 1.

***Labrum*** (not figured) ordinary. ***Paragnaths*** (= lower lip; not figured) inner lobes absent.

***Mandibles*** each with well-developed, triturative columnar molar; molar seta equally developed in both mandibles; palp lacking. ***Left mandible*** (Fig. 2A) incisor 7-denticulate; lacinia 5-denticulate. ***Right mandible*** (Fig. 2B) incisor 6-denticulate; lacinia complex, with 5 main cusps and multiple denticles, and with patch of short setules proximally on medial surface.

***Maxilla 1*** (Fig. 2C) inner lobe finger-like, crowned with two pappose setae; outer lobe with nine serrated stout robust setae distally; palp reduced, unsegmented, reaching to less than half distance between its insertion and distal margin of inner lobe, tapering with tiny conical robust seta on tip.

***Maxilla 2*** (Fig. 2D) inner lobe with two unequal pappose setae proximally on inner margin, proximal-most seta largest, hyperthophied.

***Maxilliped*** ordinary; basal endite (= inner plate) (Fig. 2E) subrectangular with three flattened triangular short cuspidate robust setae distally and row of pappose setae along medial margin.

***Coxal gills*** (Figs 3E; 4A–D) on gnathopod 2 to pereiopod 6, smooth, unstalked, sac-like. *Sternal gills* on pereionites II to VII, finger-like (Fig. 4F), placed antero-laterally at each side on corresponding sternite.

***Gnathopod 1*** (Fig. 3A–D) subchelate. Carpus longer than propodus. Propodus 1.5 times as long as broad, with concave posterior margin; palm margin evenly convex; palm angle with two short flagellate robust setae subequal in length on medial side (Fig. 3C). Dactylus with single triangular denticle subdistally on medial margin (Fig. 3D).

***Gnathopod 2*** (Fig. 3E–G) subchelate. Propodus massive, broadly expanded, 1.3 times as long as broad with palm length longer than carpal lobe; palm margin longer than posterior margin of segment, convex but sinuous, with continuous row of densely set robust setae along both sides of margin (only row on medial side shown in Fig. 3G); palm angle with two short, reduced flagellate robust setae on medial side. Unguis completely incorporated into dactylus (Fig. 3G).

***Pereiopods 3–4*** (Fig. 4A, B) similar except for coxal plates. Pereiopod 4 slightly shorter than pereiopod 3, with coxa deeply excavated posteriorly.

***Pereiopods 5–7*** (Fig. 4C–E) progressively longer towards posterior. Basis of pereiopod 7 broadest, 1.2 times as long as broad; basis of pereiopod 5 1.3 times as long as broad; basis of pereiopod 6 more slender, 1.5 times as long as broad. All limbs with propodus distinctly longer than their respective carpus.

***Pleopods*** (Fig. 1D) all similar, biramous, rami multi-annulated and provided with long plumose setae; integument of posterior face of protopod (= peduncle) and proximomedial margin of proximal article of endopod, setulose, as figured.

***Uropod 1*** (Fig. 1E) protopod longer than rami. Armature of protopod consisting of series of 3-4 flagellate robust setae proximally along posterolateral margin, and single flagellate robust seta at each posterodistal and posteromedial angle of segment; posteromedial margin of segment unarmed. Exopod with series of five flagellate robust setae along lateral margin and three robust setae on tip, none of which flagellate; medial margin of segment unarmed. Endopod with four flagellate robust setae along medial margin and three robust setae on tip, none of which flagellate; lateral margin of segment with series of four flagellate robust setae disposed subdistally on margin, of which proximal-most modified, longer than rest, slightly surpassing tip of endopod, slightly curved and complexly denticulate on tip (“copulatory spine” *sensu* Bousfield 1986; Fig. 1F).

***Uropod 2*** (Fig. 1G) protopod about as long as rami. Protopod with three flagellate robust setae along posterolateral margin; robust seta present on each posterodistal and posteromedial angle of segment, of which that on posteromedial angle non-flagellate and more slender than counterpart; posteromedial margin of segment unarmed. Exopod with five flagellate robust setae along lateral margin and three robust setae on tip; medial margin unarmed. Endopod with 3-4 flagellate robust setae along medial margin, three robust setae on tip, and three flagellate robust setae disposed subdistally on lateral margin.

***Uropod 3*** (Fig. 1H) ramus about as long as protopod. Protopod with transverse comb of 3-6 flagellate robust setae on distolateral angle, simple seta on distomedial angle, and isolated reduced simple seta on medial margin. Ramus 2.2 times longer than broad, with short robust seta and a bunch of long simple setae on tip.

***Telson*** (Fig. 1I) broader than long, with distal margin evenly rounded. Armature consisting of continuous marginal series of 4–7 (exceptionally only three) flagellate robust setae distally plus three tiny plumose setae disposed distolaterally at each side.

####### Description of brooding female.

Differing from male in smaller size (body up to 6.5 mm long); presence of oöstegites on pereionites II-V (Fig. 5A, B); aspect of gnathopod 2 (Fig. 5A), which is similar to male gnathopod 1 but with a more slender propodus (1.7 times as long as broad and attaining 95% length of carpus; versus 1.5 times as long as broad and attaining 89% length of carpus in male gnathopod 1); unequal length of robust setae on palm angle of gnathopod 2 (Fig. 5C); and endopod of uropod 1 with two flagellate robust setae along distolateral margin (versus four in male), of which none modified (Fig. 5D).

## Discussion

Of the approximately 70 species of *Hyalella* currently recognized (Horton and Lowry 2013), only nine (listed below) share a smooth, non-processiferous body, a male uropod 1 with a modified curved robust seta on endopod, and six pairs of sternal gills with *H. cajasi* sp. n. Nevertheless, the new taxon can be differentiated from each of them based on the presence/absence of eyes; relative length of antenna 1 with respect to antenna 2; presence/absence of a short pointed robust seta distally on palp of maxilla 1; number of pappose setae proximally on medial margin of inner lobe of maxilla 2; elongation and curvature of the modified robust seta on endopod of male uropod 1; relative length of ramus of uropod 3 with respect to protopod; and armature and outline of telson, among other features.

Thus, *Hyalella cajasi* sp. n. differs from *H. bonariensis* Bond-Buckup, Araujo & Santos, 2008, described in Santos et al. (2008) and known only from the province of Buenos Aires in Argentina, by the telson outline, which is broader than long and with the distal margin evenly rounded (versus telson as long as broad, subquadrate); by the telson marginal armature, arranged as a single row of robust setae (versus a cluster of robust setae distolaterally at each side); the sparsely setulose condition of the palp of maxilla 1 (versus palp densely setulose); and by the condition of the modified robust seta on the endopod of male uropod 1, which is hardly curved and only just surpasses the tip of endopod (versus seta elongate and strongly bowed).

Diagnostic differences between *H. cajasi* sp. n. and *H. brasiliensis* Bousfield, 1996, from Paraná State (Brazil), include the short ramus of uropod 3, which is shorter than the corresponding protopod (versus ramus longer than protopod); the comparatively shorter propodus of male gnathopod 1, which is shorter than carpus (versus propodus about as long as carpus); and the male gnathopod 2 propodus with palm margin longer than the posterior margin (versus palm margin about as long as posterior margin) (see Bousfield 1996).

The new species differs from *H. carstica* Bastos-Pereira & Bueno, 2012, a taxon known from Minas Gerais (Brazil), by the relative length of antenna 1, which is much shorter than antenna 2 (versus antenna 1 and antenna 2 about equal in length in *H. carstica*); by the presence of two pappose setae proximally on the medial margin of inner lobe of maxilla 2 (versus only one seta); by the broader than long telson (versus telson longer than broad); by the marginal armature of robust setae on telson, comprising a distal continuous series (versus single robust seta subdistally at each side); and by the much stouter male gnathopod 2 propodus, about 1.3 times as long as broad (versus 1.6 times, with palm margin much shorter than posterior margin) (see Bastos-Pereira and Bueno 2012).

*Hyalella cajasi* sp. n. differs from *H. castroi* González, Bond-Buckup & Araujo, 2006, a species from Rio Grande do Sul (Brazil), in the distal robust seta present on palp of maxilla 1 (versus palp pointed but devoid of armature in *H. castroi*); in the display of two pappose setae proximally on the medial margin of inner lobe of maxilla 2 (versus only one seta); in the condition of the modified robust seta on male uropod 1 endopod, which is hardly curved and only just surpasses the tip of endopod (versus seta elongate and strongly bowed); and in the stouter condition of male gnathopod 2 propodus, which is about 1.24 times as long as broad (versus 1.32 times) (see González et al. 2006).

*Hyalella curvispina* Shoemaker, 1942, known from Montevideo (Uruguay) and Rio Grande do Sul (Brazil), differs from the new species in the longer-than-broad telson, which in addition displays only 1+1 robust setae on distal margin, and the unarmed but pointed palp of maxilla 1. Furthermore, the modified setae on male uropod 1 endopod (sometimes it displays two instead of only one) are more elongate and curved than in *H. cajasi* sp. n. (see Shoemaker 1942).

*Hyalella formosa* Cardoso & Araujo, 2014 (described in Cardoso et al. 2014), from a cave in Paraná State (Brazil), differs from the new species in being eyeless; in the elongation of antenna 1, which is longer than antenna 2; in the telson armature, reduced to two terminal simple setae; and in the large size of one of the two robust setae present on palm angle of propodus of gnathopod 1.

The new species differs from *H. paramoensis* Andres, 1988, a species known from a high altitude lake near Bogotá (Colombia), in the armature of the telson, which consists of a distal series of marginal robust setae (versus armature reduced to two long and slender robust setae in *H. paramoensis*); the presence of a robust seta on tip of the palp of maxilla 1 (versus palp unarmed); and in the relative length of the modified robust seta present on the endopod of male uropod 1 (versus seta extremely elongated in *H. paramoensis*) (see Andres 1988).

*Hyalella cajasi* sp. n. differs from *H. xakriaba* Bueno & Araujo, 2013 (described in Bueno et al. 2013) from Minas Gerais (Brazil), by the palp of maxilla 1 provided with a terminal robust seta (versus seta absent); by the armature of telson (reduced to 2+2 robust setae in *H. xakriaba*); and by the stouter male gnathopod 2 propodus (1.24 times as long as broad; versus 1.59 times in *H. xakriaba*).

Finally, *H. cajasi* sp. n. differs from *H. veredae* Cardoso & Bueno, 2014 (described in Cardoso et al. 2014), known from a cave in Minas Gerais (Brazil), by the non-regressed, ordinary eyes (versus eyes reduced or absent); by the relative length of antenna 1 (shorter than antenna 2; versus antenna 1 and antenna 2 about equal in length); and by the armature and outline of telson, subquadrate and with only 1+1 robust setae in *H. veredae*.

With regards to *H. meinerti*, the only representative of the genus confidently known to occur in Ecuador until now, it differs from *H. cajasi* sp. n. in the display of a non-sexually dimorphic uropod 1, a 1+1 armature arrangement on distal margin of the telson, and in the presence of only one pappose seta proximally on the medial margin of the inner lobe of maxilla 2.

## Supplementary Material

XML Treatment for Hyalella cajasi
